# Prospective Real-World Gynaecological Cancer Clinical Registry with Associated Biospecimens: A Collaborative Model to Promote Translational Research between GEICO and the Spanish Biobank Network

**DOI:** 10.3390/cancers14081965

**Published:** 2022-04-13

**Authors:** José Antonio López-Guerrero, Marta Mendiola, José Alejandro Pérez-Fidalgo, Ignacio Romero, Ana Torres, Delia Recalde, Elena Molina, César Gómez-Raposo, Ana M. Levin, Ana Herrero, Jesús Alarcón, Carmen Esteban, Gloria Marquina, María Jesús Rubio, Eva Guerra, Luisa Sánchez-Lorenzo, Fernando Gálvez-Montosa, Ana de Juan, Cristina Churruca, Alejandro Gallego, Antonio González-Martín

**Affiliations:** 1 Laboratorio de Biología Molecular y Biobanco, Fundación Instituto Valenciano de Oncología, 46009 Valencia, Spain; 2 Unidad Mixta de Investigación en Cáncer IVO-CIPF, Centro de Investigación Príncipe Felipe (CIPF), 46012 Valencia, Spain; 3 Departamento de Patología, Facultad de Medicina, Universidad Católica de Valencia ‘San Vicente Martir’, 46001 Valencia, Spain; 4 Laboratorio de Patología Molecular y Dianas Terapéuticas, Instituto de Investigación Hospital Universitario La Paz (IdiPAZ), 28029 Madrid, Spain; marta.mendiola@gmail.com; 5 Centro de Investigación Biomédica en Red de Cáncer, CIBERONC, Instituto de Salud Carlos III, 28029 Madrid, Spain; 6 Departamento de Oncología Médica, Hospital Clinico de Valencia, Instituto de Investigación Sanitaria INCLIVA, Universidad de Valencia, CIBERONC, 46010 Valencia, Spain; jopefi@uv.es; 7 Department of Medical Oncology, Fundación Instituto Valenciano de Oncología, 46009 Valencia, Spain; iromero@fivo.org; 8 Biobanco del Hospital Universitario Ramón y Cajal, 28034 Madrid, Spain; atredondo@salud.madrid.org; 9 Biobanco del Sistema de Salud de Aragón, 50009 Zaragoza, Spain; drecalde.iacs@aragon.es; 10 Biobanco del Hospital Clínico San Carlos, 28040 Madrid, Spain; elenamilagrosa.molina@salud.madrid.org; 11 Department of Medical Oncology, Hospital Universitario Infanta Sofía, 28703 Madrid, Spain; cgomezr@salud.madrid.org; 12 Grupo Español de Investigación en Cáncer de Ovario, 28003 Madrid, Spain; alevin@grupogeico.net; 13 Department of Medical Oncology, Hospital Universitario Miguel Servet, 50009 Zaragoza, Spain; aherreroi@salud.aragon.es; 14 Department of Medical Oncology, Hospital Universitario Son Espases, 07120 Palma, Spain; jesus.alarcon@ssib.es; 15 Department of Medical Oncology, Hospital Virgen de la Salud, 45004 Toledo, Spain; cesteban@sescam.jccm.es; 16 Department of Medical Oncology, Hospital Clínico San Carlos, 28040 Madrid, Spain; gloria.marquina@salud.madrid.org; 17 Department of Medical Oncology, Hospital Universitario Reina Sofía, 14004 Córdoba, Spain; mjesusrubio63@gmail.com; 18 Department of Medical Oncology, Hospital Universitario Ramón y Cajal, 28034 Madrid, Spain; evamaria.guerra@salud.madrid.org; 19 Department of Medical Oncology, Clínica Universidad de Navarra, 28027 Madrid, Spain; lsanchezl@unav.es (L.S.-L.); agonzalezma@unav.es (A.G.-M.); 20 Department of Medical Oncology, Hospital Universitario de Jaén, 23007 Jaén, Spain; fernando.galvez.sspa@juntadeandalucia.es; 21 Department of Medical Oncology, Hospital Univeristario Marqués de Valdecilla, 39008 Santander, Spain; anade.juan@scsalud.es; 22 Department of Medical Oncology, Hospital Universitario Donostia, 20014 Donostia, Spain; cristinamaria.churrucagalaz@osakidetza.eus; 23 Department of Medical Oncology, Hospital Universitario La Paz, 28029 Madrid, Spain; alejandro.gallego@salud.madrid.org; 24 Programa de Tumores Sólidos, Centro de Investigación de Medicina Aplicada (CIMA), 31008 Pamplona, Spain

**Keywords:** patient registry, biobanking, biospecimens, gynaecological cancer, harmonisation, translational research

## Abstract

**Simple Summary:**

This is a collaborative approach between two multicentre structures, the Spanish Group of Ovarian Cancer Research (GEICO) and the Spanish Biobank Network (RNBB), for collecting high-quality clinical data and associated biospecimens from gynaecological patients in the context of a virtual clinical registry (VCR). The model described herein defines the mode of operation of this initiative and represents a win-win example between multicentre structures that will contribute to the success of translational research in gynaecological cancer. As of December 2021, 30 active sites have recruited a total of 655 patients in the GEICO VCR, distributed in the following cohorts: Ovarian Cancer (377), Endometrial Cancer (206), Cervical Cancer (67), and Rare Tumours (5). The main type of associated biospecimens are formalin-fixed and paraffin-embedded tissue, plasma, serum, and buffy-coat.

**Abstract:**

Patient registries linked to biorepositories constitute a valuable asset for clinical and translational research in oncology. The Spanish Group of Ovarian Cancer Research (GEICO), in collaboration with the Spanish Biobank Network (RNBB), has developed a multicentre, multistakeholder, prospective virtual clinical registry (VCR) associated with biobanks for the collection of real-world data and biological samples of gynaecological cancer patients. This collaborative project aims to promote research by providing broad access to high-quality clinical data and biospecimens for future research according to the needs of investigators and to increase diagnostic and therapeutic opportunities for gynaecological cancer patients in Spain. The VCR will include the participation of more than 60 Spanish hospitals entering relevant clinical information in harmonised electronic case report forms (eCRFs) in four different cohorts: ovarian, endometrial, cervical, and rare gynaecological cancers (gestational trophoblastic disease). Initial data for the cases included till December 2021 are presented. The model described herein establishes a real-world win-win collaboration between multicentre structures, promoted and supported by GEICO, that will contribute to the success of translational research in gynaecological cancer.

## 1. Introduction

Clinical registries, understood as electronic databases used to collect clinical data in observational studies, enable the evaluation of patient characteristics and clinical outcomes in a real-world setting [1,2]. Cancer patient registries typically include demographics, medical history, tumour diagnosis, treatment, radiological response, and survival information. In the context of collaborative gynaecological cancer research, academic groups use clinical registries to gain insights into the clinical evolution of specific tumour types, monitor their management and outcomes, and develop research hypotheses to improve diagnosis and the design of new therapies [3]. The development of personalised treatment approaches, based on the understanding of tumour biology and the identification of biomarkers, requires the correlation of clinical data and the molecular information obtained from a patient’s samples (mainly tumour tissue and blood) [4]. Thus, the integration of clinical registries with biobank material becomes essential to gain a better knowledge of the disease and the development of new therapeutic options [5].

Biobanks are highly structured institutional units that host collections of human biological material (biospecimens) linked to relevant personal, health, genetic, and genomic information, with strictly regulated access [6]. In oncology, biobanks store tumour tissue, blood, and other fluids to be used in clinical and translational research, with prior patients’ consent for collected material storage and research use [7]. Common sources of biological samples include diagnostic biopsies and surgeries in standard clinical practice as well as non-standard extractions in clinical trials [8]. Biobanks provide an invaluable resource to medical oncologists and molecular biologists involved in clinical research so that improved and wider access to these biorepositories with accompanying high-quality clinical data becomes a strategic priority for current and future cancer research projects [9].

The limitations of individual biobanks to support research studies have led to the recognition that cooperation through networks, including multiple biobanks, is more productive than individual strategies [10], a trend that is increasing internationally [11,12,13]. Biobank networking promotes the sharing of tools and experience for better data interconnectivity and reutilisation, as well as harmonised procedures and best practices for biological sample management, which increases the quality of biospecimens for research purposes [11].

The Spanish Ovarian Cancer Research Group (Grupo Español de Investigación en Cancer de Ovario—GEICO) has led clinical and translational gynaecological cancer research in Spain since 1999, gathering more than 131 hospitals and 334 multidisciplinary researchers as of December 2021. GEICO seeks to improve outcomes for women with gynaecological cancer through quality research, facilitation of clinical trials, and best practice in clinical care and has become an active member of other international collaborative groups such as the European Network of Gynaecological Oncological Trial Group (ENGOT) and the Gynaecologic Cancer Intergroup (GCIG). As a result of these collaborations, numerous clinical trials have been conducted to change the paradigm of gynaecological cancer treatment, with significant recent examples in ovarian [14,15] and cervical cancer [16].

As a result of its multidisciplinary character and its participation in multiple clinical trials with very well annotated information, GEICO started a win-win collaboration with the Spanish Biobank Network (Red Nacional de BioBancos—RNBB) to develop a multicentre, multistakeholder, prospective virtual clinical registry (VCR) associated with biobanks for the collection of real-world data and biological samples of gynaecological cancer patients. The project seeks to increase knowledge of the reality and management of these patients in Spain, to gain insights into the improvement of diagnosis and therapeutic options, and to provide broad access to high-quality clinical data and biospecimens for future research according to the needs of investigators.

## 2. Materials and Methods

### 2.1. The GEICO VCR Design

A stepwise, multidisciplinary, highly collaborative, and goal-oriented approach was taken to create a robust GEICO VCR (GEICO 81-T study) based on a harmonised web-based electronic case report form (eCRF) system.

The registry represents an observational, prospective, and multicentre study with a national scope of more than 60 hospitals that voluntarily decide to collaborate by entering relevant clinical information in four different cohorts: OC, endometrial (EC), cervical (CC), and rare cancers (RC). Inclusion criteria to participate in the VCR are women diagnosed with gynaecological cancer after the activation of each centre; age ≥ 18 years; written informed consent; and access to source medical records. The VCR collects data obtained from medical records used in routine clinical practice, including clinical-pathological characteristics, evolution, and management of gynaecological cancer patients. All this information is electronically stored in 7 datasets: Patient Data (anonymised); diagnosis; primary surgery; chemotherapy; biobank; recurrence; and follow-up (Table 1). The study protocol and the patient information sheet and informed consent form (PIS/ICF) were approved by the required ethics committees (ECs) of the participating sites in Spain (first approval on 15 May 2019).

### 2.2. The GEICO VCR Development Strategy

**Figure 1:**
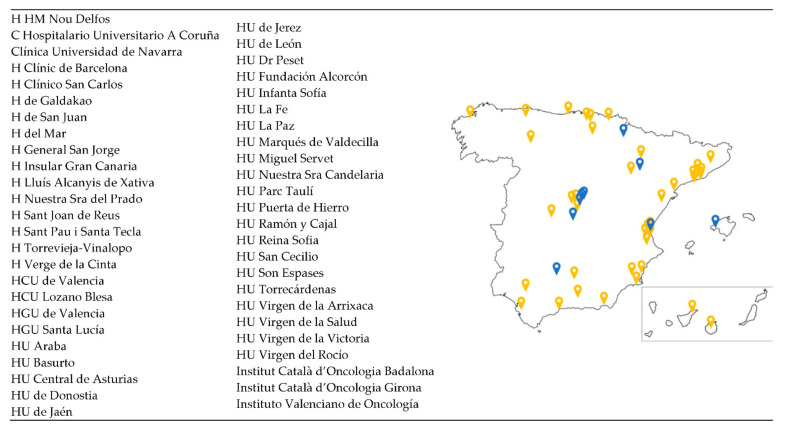
Distribution of the GEICO centres involved in the VCR. A total of 49 centres distributed throughout Spanish territory are participating in the VCR. Those centres labelled in blue were involved in the pilot study; the yellow ones were added in successive phases.

Between June and November 2019, nine Spanish hospitals belonging to seven different autonomous communities participated in a pilot study for the launch of the VCR with the OC module only. Additional sites have been added in subsequent phases, and currently (December 2021), a total of 49 sites are involved in Spain (30 fully activated and 19 in process) (Figure 1). Research teams at clinical sites (medical oncologists and study coordinators) obtain the informed consent from patients and then proceed to enter the clinical data in the web-based eCRF.

### 2.3. Clinical Data and Biological Sample Collection Process

**Figure 2:**
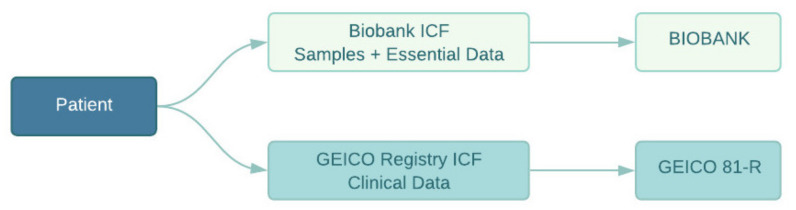
Patients sign two ICFs: one corresponding to the VCR, allowing the incorporation of clinical data to the web-based application; and one to the biobank ICF for the collection of biospecimens and minimal data.

The patient’s clinical data and biological sample collection process follow a two-fold path (Figure 2). On the one hand, the clinical principal investigator (medical oncologist) interviews the patient during a hospital visit, explaining the details of the study and providing a PIS/ICF to be read and signed by the patient. Once the patient consents to participation, the investigator or a member of his/her research team will enter the clinical data of the patient in the VCR. On the other hand, and not necessarily at the same time, the biological samples of the patient, along with a minimum set of associated data, are stored at the biobank once the patient has signed the biobank ICF.

All participating sites have an associated biobank; most of them are full or associated members of RNBB. The type of biospecimen collected in each biobank was decided by the site research team in agreement with the biobank manager, considering collection capabilities. As shown in Table 2, collected samples include formalin-fixed and paraffin-embedded (FFPE) and fresh-frozen tissue, serum, plasma EDTA, buffy coat, peritoneal lavage, and others.

### 2.4. Biobank Network Mode of Operation

**Figure 3:**
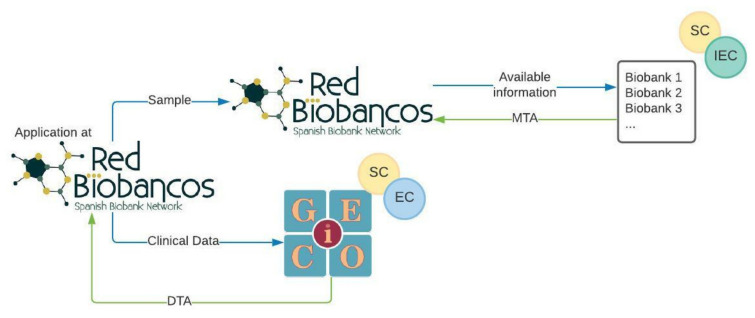
Single access point model to obtain clinical data and biological samples. Application for samples and data is performed through the RNBB. SC: Scientific Committee, IEC: Independent Ethics Committee, EC: Executive Committee, MTA: Material Transfer Agreement, DTA: Data Transfer Agreement.

A policy to access the clinical data and biospecimens from patients included in the VCR was defined by GEICO and the RNBB based on a single window model (Figure 3).

An investigator interested in using the biospecimens for gynaecological cancer research applies to the RNBB through specific forms. In these questionnaires, the applicant indicates the criteria for selecting the biospecimens, number, tumour type, type of sample (FFPE, blood, serum, plasma, etc.), and type of clinical information needed. The Coordination Office of the RNBB forwards the application to the involved biobanks (members and associated), and each biobank elaborates an availability report with the samples that meet the selection criteria of the application. Once the RNBB confirms the feasibility of the request, the investigator must send the documents related to the research project so that the biospecimen request can be evaluated by the ethics and scientific committees associated with each biobank, as established by the Spanish regulation [6]. Upon authorisation, the biobanks send the biospecimens to the investigator together with a Material Transfer Agreement (MTA) establishing the conditions and relationships between the investigator and each biobank. This could also include economical revenues for biospecimen handling and management. In parallel, the Coordination Office of the RNBB communicates to GEICO the interest of the investigator in having access to the clinical data of the VCR corresponding to the requested samples. This application will be assessed by GEICO’s Scientific Committee and Executive Board. Once the application is approved, the clinical data are shared together with a Data Transfer Agreement (DTA) in which the particularities of the collaboration between the investigator and GEICO are defined. A specific working group, including key members from the RNBB and GEICO, has been appointed to monitor all the applications involving biospecimens and clinical data from patients included in the VCR.

### 2.5. Commitment to Patients

In order to be aligned with the interests of patients with gynaecologic cancer, GEICO contacted the Association of People Affected by Ovarian Cancer (ASACO). ASACO’s mission is to support OC patients, relatives, and caregivers, providing them with personalised advice in social, medical, and legal aspects. This includes information about new research and medical advances related to gynaecological cancer. GEICO explained the VCR protocol, including the type of collected clinical information and the opportunity to participate by providing biological sample remainders from diagnostic processes to the biobanks for further research. Furthermore, ASACO members assisted in defining the PIS/ICF content to participate in the VCR. As per ASACO’s initiative, a Google form survey asking about patients’ expectations and priorities with regard to gynaecological cancer research was sent to the association’s members.

## 3. Results

### 3.1. VCR Recruitment and Clinical Data

As of December 2021, 30 active sites recruited a total of 655 patients in the GEICO VCR, including real-world data distributed in the following cohorts: OC (377), EC (206), CC (67), and Rare Tumours (5). For the OC cohort, age groups, diagnosis, clinical trial involvement, FIGO stage, HRD alteration, primary surgery, and chemotherapy data are presented in Table 3 and Table 4.

### 3.2. Model Operating Policy

A set of premises were defined to ensure the proper functioning of the model presented herein:

1. Participation in the GEICO VCR is voluntary. All GEICO centres and researchers were invited to join the VCR, but only those who expressed their interest in participating were activated. To date, there are 49 centres involved (30 fully activated for recruitment). The target is to activate at least a total of 60 sites;
2. Both GEICO and RNBB maintain their independence in terms of functioning. This is the reason why patients sign two informed consents: the one to provide clinical data for the VCR and the one to give biospecimens for the biobank. This implies that there might be patients who provide data without samples and vice versa;
3. Each centre coordinates the collection of data and biospecimens. Each local GEICO investigator coordinates with the biobank manager the type of biological samples to be collected according to the biobank’s capabilities. At least an FFPE tumour block is recommended;
4. Biobanks will charge investigators the cost of handling the samples to cover expenses. This will be done for each application and will affect only those sample types that are selected. The invoice for this service is provided once the samples are delivered to the investigator. Biobanks might apply different fees depending on the type of applicant (public, private, pharma, etc.) and are agreed with the investigator prior to signing the MTA;
5. GEICO will not receive any financial income for providing data. The only expected return for GEICO is of scientific and academic nature. GEICO has developed a publication policy to compensate the efforts of investigators in maintaining the VCR updated, which is mainly based on co-authorships for publications originating from the results of the investigations. This aspect is discussed with the investigator and defined in the DTA.

### 3.3. Patient Participation

**Figure 4:**
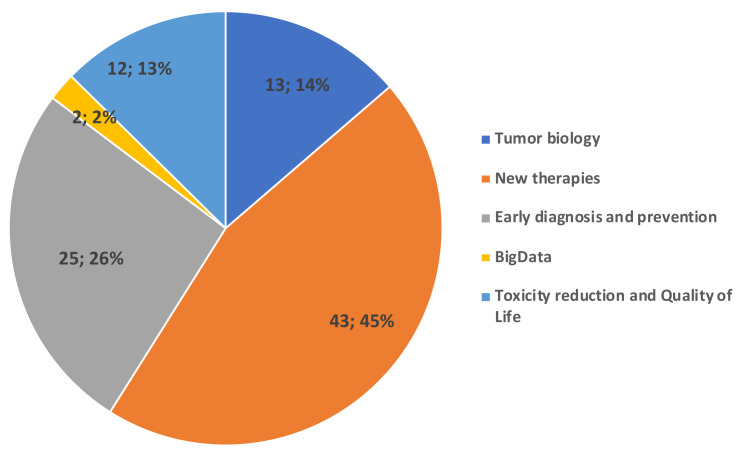
Main research interests of ASACO patients were new therapies and early diagnosis and prevention.

The enthusiastic interest of ASACO towards the VCR, and their involvement in supporting GEICO, motivates gynaecological cancer patients to be enrolled in the registry. To this end, ASACO funded and participated in the design of an information brochure (Supplementary information) that is provided to patients together with the ICFs. This brochure provides information about the following items: the VCR’s purpose; GEICO; what is a clinical registry; what can be done with the registry; what is translational research; what are their benefits; and how they can participate. At the same time, ASACO wanted to motivate GEICO investigators by showing patients’ priorities in cancer research through the results of a voluntary survey shared among their associates. This type of survey might contribute to the decision-making process for prioritising research associated with the GEICO VCR. A total of 87 surveys were collected from women with a median age of 53 years (range: 28–70 yrs) who have suffered from OC diagnosed between 1992 and 2019. The results from the survey showed that investigation of new treatments (including immunotherapy) (45%) and prevention and early diagnosis (26%) constituted the research priorities for patients. The reduction of toxicities and Quality of Life, and tumour biology were the following areas of interest with a frequency of 13% and 14%, respectively (Figure 4).

## 4. Discussion

Patient registries linked to biorepositories constitute a tremendously valuable asset for clinical and translational research in oncology, particularly in the emerging area of personalised medicine. The advent of precision medicine in cancer therapeutics requires biobanks to provide the adequate biological material needed to investigate the complex nature of cancer [17]. Therefore, the availability of harmonised and accessible clinical data registries affiliated with biobanks has become an essential driver to improve the outcomes of future collaborative cancer research.

Recent studies related to gynaecological cancer biobanking and data harmonisation, including living biobanks of ex vivo cultures of OC models [18]; the Canadian Ovarian Experimental Unified Resource (COEUR) [19] to provide access to biological material and data for biomarker research in OC; the BRandOBio [20] large multicentre translational project for newly diagnosed OC patients with affiliated biobank; the prospective Australian National Gynae-Oncology Registry (NGOR) [3]; the development of tools for harmonisation of biobanking standards in endometrial cancer research (HASTEN study) [21]; and The Cancer Genome Atlas (TCGA) project, are further discussed below.

### 4.1. The Importance of High-Quality Clinical Data and Biospecimens

The GEICO VCR is presented as a tool for collecting clinicopathological information of great value to know the reality of patients diagnosed with gynaecological cancers in Spain, particularly their prevalence, evolution, and general management. The project aims to promote research by providing broad access to high-quality clinical data and biospecimens for future research according to the needs of investigators and to increase diagnostic and therapeutic opportunities for gynaecological cancer patients in Spain.

GEICO has been a pioneer in Spain in the field of clinical databases for research purposes, and in 1998, the group established an early-stage OC registry that now includes more than 1100 patients [22]. From this registry, international relations have been established, such as that with the Massachusetts General Hospital (Boston, MA, USA), with which GEICO participates in TCGA project, contributing with cases and associated clinical information, which has allowed a deeper knowledge about the molecular basis of OC early stages [23].

Clinical research with biospecimens has existed for more than 50 years, initially as particular collections with a very local use [24]. In the late 1980s, biobanks started to emerge due to a growing demand for biological samples and data, significantly motivated by the irruption of biomarker research. However, few biobanks focused their activity on ensuring the quality of biological samples and meeting the specific needs of researchers. It was at the beginning of TCGA project when some biological samples stored in biobanks were considered “inadequate” [24,25]. This episode illustrates that many biospecimens have been globally collected with poor quality control (QC) mechanisms and without considering the specific needs of researchers, and they may therefore never be used.

### 4.2. The TCGA Experience

For the past decade, oncology research has undergone an exciting transformation, driven by advances in genomic technologies and reinforced by international collaborations. The best example is represented by TCGA, a comprehensive project started in 2005 to increase the knowledge of the molecular basis of cancer through the use of genome analysis technologies, including large-scale genome sequencing [26]. From the beginning, it became evident that the success of TCGA would be based on the quality of the biological material to be analysed [25], and special efforts were made in this sense [27]. In the case of TCGA of OC, a total of 1020 matched OC cases were collected. As a whole, approximately 45% of cases failed in QC and were not enrolled in the study [27]. This experience shows that sample quality is of utmost importance, as it is well-known that “preanalytical” variables such as sampling procedures, tissue origin, and preservation factors, among others, have an impact on biospecimen analysis [28]. In addition, health-related data are crucial to increase the scientific value of biospecimens to support clinical research [10,29], in the case of cancer, particularly for biomarker research and personalised medicine [30].

As a consequence of the above example, the role of biobanks, considered as technical units for the management of high-quality biospecimens, has become crucial for guaranteeing the success of translational research. RNBB biobanks are committed to sampling quality and procedure harmonisation, which is even more critical when biobanks are organised into networks. The implementation of common Quality Assurance Management Systems in biobanks and biobank networks constitutes the key to guaranteeing the harmonisation of all processes to reduce the inter-biobank variabilities [31,32].

### 4.3. The Advantages of the VCR-Biobank Experience

Accessing and managing biological samples through the GEICO-RNBB VCR-biobank model provides highly valuable advantages. Firstly, this collaborative structure ensures high-quality biological material and clinical data, which is a critical aspect to conduct reliable and reproducible cancer research studies. Secondly, attending physicians are stimulated to collect biological material and clinical data from gynaecological cancer patients that may be used by their own research projects. Thirdly, the institutional biobank and the clinical research group (GEICO) remain autonomous, independent from each other, as they both maintain their own assets through a transparent model, in which they benefit from the joint use of shared resources. Finally, the wider availability of biospecimens with associated clinical data encourages translational research not only by GEICO investigators but also by third parties, guaranteeing the return on investment at both financial and academic levels.

### 4.4. Multidisciplinary International Collaboration: The Path towards Effective Personalised Medicine

Currently, clinical databases and biobanks are vital elements towards the effective development of personalised medicine. In fact, within the European Commission’s framework programmes, biobank involvement is required, and collaboration with registries, repositories, and research infrastructures is highly recommended [9]. According to Horizon Europe Work Programme 2021–2022, cancer project proposals should build on biobanks and registries [33]. Research infrastructures are crucial pillars to face global healthcare challenges.

Personalised medicine has emerged as an innovative scientific approach that requires a very high degree of collaboration across disciplines and nations. Clinical and translational research is eminently multidisciplinary because science has become more complex and specialised, with new research collaborations being constantly established globally [34]. Research projects are frequently global in nature, involving team members with different types of expertise, such as clinicians, molecular biologists, laboratory technicians, bioinformaticians, statisticians, and other data analysts [34].

The challenges posed by clinical data and biospecimen collection, storage, access, sharing, and harmonisation cannot be faced via isolated efforts. Clinical registries and biobanks are connected through intricate multinational networks. Therefore, for the future of clinical and translational research, a multidisciplinary approach is necessary, international cooperation is essential [35], and education and research programmes are the basic prerequisites for the personalised development of useful biobanks with accurate associated data. The GEICO-RNBB partnership seeks to serve this purpose.

Cancer patients are the main stakeholders in this type of initiative as, in the end, the results of the research will impact their benefit. For this reason, empowering patients in the design, dissemination, and communication of the model presented herein represents an added value that enriches and motivates us all. Knowing their feelings and worries helps us as researchers and GEICO as a collaborative group to prioritise the research focus in line with the patients’ needs.

### 4.5. Limitations of the Approach

Although the strategy that has been presented here tries to circumvent many of the limitations implicit in collaborative research, such as data harmonisation, biological sample quality, or equal access to samples and data, it is not, however, exempt from some of them. For instance, although the approach targets all patients with gynaecological cancer, only those who gave their consent to participate are included in the VCR, which could generate some bias when trying to extrapolate the collected information to the general population. Another limitation is that all patients registered in the VCR have associated biospecimens in the biobank, which requires close coordination between the involved parties of each centre (gynaecologists, oncologists, pathologists, and biobank). We noticed that in some of the centres, a channel of communication between these parties did not exist, particularly with the biobank, and thanks to this initiative, the collection of gynaecological biospecimens has been improved. In any case, we consider that multidisciplinary tumour boards constitute an ideal framework to coordinate the logistics for each centre.

Data collection and sharing is a limitation and a challenge at the same time. The VCR is designed to collect relevant data only, and most fields have drop-down menus to avoid inconsistencies and decrease the risk of mistakes. As a built-in quality control mechanism, the tool integrates automated queries triggered to alert users about incorrect and/or empty fields. The platform is very user-friendly and simple, so collecting the data of a patient may entail nearly 20 min on average. There is no possibility to export the minimum set of data directly from the patient electronic clinical record to the VCR, so nurturing the database depends directly on the scientific interest and commitment of the researcher of each centre. Any initiative might be considered for inclusion in the VCR, as it represents a consensual, centralised, structured, and harmonised database easily exploitable not only by individual researchers but also by other international collaborative structures of which GEICO is an active member such as GCIG or ENGOT.

## 5. Conclusions

The GEICO VCR, in association with the RNBB, provides an innovative, effective, and reproducible multidisciplinary collaboration model to facilitate access to high-quality clinical data and related biospecimens of gynaecological cancer patients in Spain. The project will not only increase the knowledge of the reality and management of these patients, eventually leading to the improvement of their diagnosis and treatments, but it will also generate better resources and tools for translational research to be conducted by future generations.

## Figures and Tables

**Table 1:** Summary of the data included in the different datasets of the GEICO VCR.

**Patient Data**	Site Patient ID Date of birth Date of registration Date of informed consent	Consent to transfer data to third parties Willingness to be informed about findings Participation in Clinical Trials
**Diagnosis**	Date of diagnosis Ovarian cancer (Epithelial malignant, Non-epithelial malignant, Borderline, Non-malignant) - Epithelial malignant (Serous, Endometrioid, Mucinous, Clear cell, Mullerian/carcinosarcoma, Other), - Differentiation grade - Non-epithelial malignant (Granulosa, Yolk sac, Germinal, Brenner, Other) - Borderline (Serous, Endometrioid, Other) - Non-malignant (Cyst, Endometriosis, Other)	Endometrial cancer (Endometrioid serous/carcinosarcoma, Sarcoma, Other) Cervical cancer (Squamous carcinoma, Adenocarcinoma, Sarcoma, Other) Secondary malignancy Germline: BRCA1/2 alterations Somatic: BRCA1/2 and HRD alterations Federation Internationale de Gynecolgie et d’Obstetrique (FIGO) stage according to 2014 classification Biopsy number
**Primary surgery**	Tumour type (Primary, Recurrent) Surgery type (Palliative, Laparoscopy, Laparotomy)	ECOG status CA-125 Residual tumour mass
**Chemotherapy**	Drug name Start and end dates	Maintenance
**Biobank**	Availability of sample in biobank Biobank code	Availability of sample in Pathological Anatomy Service
**Recurrence**	Yes/No	Recurrence dates
**Follow-up**	Date of last follow up	Death (Date, Cause)

**Table 2:** Type of biospecimens collected by local biobanks.

Biobank/Institution	Region	FFPE Block	Plasma	Serum	Buffy Coat	Ascitic Fluid	Other
HCU San Carlos	Comunidad de Madrid	✓	✓	✓	✓		
HU Son Espases	Baleares	✓					*
H U Reina Sofía	Andalucía	✓	✓	✓	✓		**
HU Virgen de la Salud	Castilla la Mancha	✓	✓	✓			*
HU Miguel Servet	Aragón	✓	✓	✓	✓		PBMC in DMSO
CUN	Navarra	✓	✓	✓	✓		*
IVO	Comunidad Valenciana	✓	✓	✓	✓	***	*
HU Ramón y Cajal	Comunidad de Madrid	✓	✓	✓	✓		*
HU Infanta Sofía	Comunidad de Madrid	✓	✓	✓	✓		
Hospital del Mar	Cataluña	✓	✓	✓	✓		
HU La Fe	Comunidad Valenciana	✓	✓	✓	✓		
H CU de Valencia	Comunidad Valenciana	✓	✓	✓	✓		*
ICO Girona	Cataluña	✓	✓	✓	✓		
HU La Paz	Comunidad de Madrid	✓	✓	✓	✓		
HU Marqués de Valdecilla	Cantabria	✓	✓	✓	✓		
CHUAC	Galicia	✓	✓	✓	✓		
Parc Taulí Hospital Universitari	Cataluña	✓	✓	✓	✓		
HU Central de Asturias	Asturias	✓	✓	✓	✓		*
ICO Badalona	Cataluña	✓	✓	✓	✓		*
HU Jaén	Andalucía	✓					
HU Virgen del Rocío	Andalucía	✓					
HU Virgen de la Victoria	Andalucía	✓					
HU Torrecárdenas	Andalucía	✓	✓	✓	✓	***	
HU de Jerez	Andalucía	✓					
HCU San Cecilio	Andalucía	✓					
H Galdakao	País Vasco	✓	✓	✓	✓		
HU Basurto	País Vasco	✓	✓	✓	✓		

FFPE, formalin-fixed paraffin-embedded; PBMC, Peripheral Blood Mononucleated Cells; DMSO, Dimethyl sulfoxide; (*) fresh-frozen tissues for some cases; (**) Fresh frozen tissue in RNAlater for some cases; (***) for some cases.

**Table 3:** Baseline characteristics of the OC cohort VCR data.

Age Group (Years)		Diagnosis			
30–50	85	Borderline		15	
51–70	204	Epithelial malignant		307	
17–90	85	Non-epithelial malignant		2	
**ECOG status (initial diagnosis)**		**FIGO stages**			
0	158	IA IB IC IC1 IC2 IC3 IIA IIB	36 4 5 19 12 9 11 11	IIA IIIA1 IIIA2 IIIB IIIC IVA IVB	11 10 2 14 111 16 62
1	97				
2	15				
3	1				
4	2				
NA	24				
**HRD alteration**					
Yes	7				
No	5				
Unknown	82				

HRD: Homologous recombination deficiency NA: Not available.

**Table 4:** Previous surgeries and treatments of the OC cohort VCR data.

Clinical Trial Involvements			
Yes	44	No	323
**Primary tumour surgery**		**Primary tumour surgery (technique)**	
Diagnostic (only biopsy) Interval debulking Palliative Primary cytoreduction Secondary cytoreduction NA	41 55 4 183 1 13	Laparoscopy	60
		Laparotomy	198
		NA	39
		**Residual tumour mass (primary) [cm]**	
		>1	46
		0	184
		0.1–1	20
		NA	47
**Chemotherapy**			
Bevacizumab	41	Paclitaxel	222
Carboplatin	258	PLD	13
Cisplatin	1	Others	23
Gemcitabine	1		

PLD: Pegylated liposomal doxorubicin.

## Data Availability

Data are available on reasonable request from authors.

## References

[1] Husson O., de Rooij B.H., Kieffer J., Oerlemans S., Mols F., Aaronson N.K., van der Graaf W.T.A., van de Poll-Franse L.V. (2020). The EORTC QLQ-C30 Summary Score as Prognostic Factor for Survival of Patients with Cancer in the ‘Real-World’: Results from the Population-Based PROFILES Registry. Oncologist.

[2] Zanetti R., Sacchetto L., Coebergh J.W., Rosso S. (2018). To accelerate cancer prevention in Europe: Challenges for cancer registries. Eur. J. Cancer.

[3] Heriot N., Brand A., Cohen P., Hegarty S., Hyde S., Leung Y., Zalcberg J.R., Rome R. (2020). Developing an Australian multi-module clinical quality registry for gynaecological cancers: A protocol paper. BMJ Open.

[4] Ringborg U. (2019). Translational cancer research—A coherent cancer research continuum. Mol. Oncol..

[5] Roberts J.N., Karvonen C., Graham K., Weinfeld M., Joy A.A., Koebel M., Morris D., Robson P.J., Johnston R.N., Brockton N.T. (2015). Biobanking in the Twenty-First Century: Driving Population Metrics into Biobanking Quality. Adv. Exp. Med. Biol..

[6] Arias-Diaz J., Martín-Arribas M.C., del Pozo J.G., Alonso C. (2013). Spanish regulatory approach for biobanking. Eur. J. Hum. Genet..

[7] Caenazzo L., Tozzo P., Borovecki A. (2015). Ethical governance in biobanks linked to electronic health records. Eur. Rev. Med. Pharmacol. Sci..

[8] Annaratone L., De Palma G., Bonizzi G., Sapino A., Botti G., Berrino E., Mannelli C., Arcella P., Di Martino S., Steffan A. (2021). Basic principles of biobanking: From biological samples to precision medicine for patients. Virchows Arch..

[9] Kinkorová J., Topolčan O. (2018). Biobanks in Horizon 2020: Sustainability and attractive perspectives. EPMA J..

[10] Riegman P.H.J., Morente M.M., Betsou F., de Blasio P., Geary P. (2008). Biobanking for better healthcare. Mol. Oncol..

[11] Meir K., Gaffney E.F., Simeon-Dubach D., Ravid R., Watson P.H., Schacter B., Manuel M., Morente and the Marble Arch International Working Group on Biobanking (2011). The human face of biobank networks for translational research. Biopreserv. Biobank..

[12] Morente M.M., Cereceda L., Luna-Crespo F., Artiga M.J. (2011). Managing a biobank network. Biopreserv. Biobank..

[13] Devereux L., Watson P.H., Mes-Masson A.-M., Crespo F.D.L., Thomas G., Pitman H., Speirs V., Hall A.G., Bollinger N., Posada M. (2019). A Review of International Biobanks and Networks: Success Factors and Key Benchmarks-A 10-Year Retrospective Review. Biopreserv. Biobank..

[14] González-Martín A., Pothuri B., Vergote I., DePont Christensen R., Graybill W., Mirza M.R., McCormick C., Lorusso D., Hoskins P., Freyer G. (2019). Niraparib in Patients with Newly Diagnosed Advanced Ovarian Cancer. N. Engl. J. Med..

[15] Ray-Coquard I., Pautier P., Pignata S., Pérol D., González-Martín A., Berger R., Fujiwara K., Vergote I., Colombo N., Mäenpää J. (2019). Olaparib plus Bevacizumab as First-Line Maintenance in Ovarian Cancer. N. Engl. J. Med..

[16] Tewari K.S., Sill M.W., Long H.J., Penson R.T., Huang H., Ramondetta L.M., Landrum L.M., Oaknin A., Reid T.J., Leitao M.M. (2014). Improved survival with bevacizumab in advanced cervical cancer. N. Engl. J. Med..

[17] Hewitt R.E. (2011). Biobanking: The foundation of personalized medicine. Curr. Opin. Oncol..

[18] Nelson L., Tighe A., Golder A., Littler S., Bakker B., Moralli D., Baker S.M., Donaldson I.J., Spierings D.C.J., Wardenaar R. (2020). A living biobank of ovarian cancer ex vivo models reveals profound mitotic heterogeneity. Nat. Commun..

[19] Le Page C., Rahimi K., Köbel M., Tonin P.N., Meunier L., Portelance L., Bernard M., Nelson B.H., Bernardini M.Q., Bartlett J.M.S. (2018). Characteristics and outcome of the COEUR Canadian validation cohort for ovarian cancer biomarkers. BMC Cancer.

[20] De Gregorio A., Nagel G., Möller P., Rempen A., Schlicht E., Fritz S., Flock F., Kühn T., Thiel F., Felberbaum R. (2020). Feasibility of a large multi-center translational research project for newly diagnosed breast and ovarian cancer patients with affiliated biobank: The BRandO biology and outcome (BiO)-project. Arch. Gynecol. Obstet..

[21] Adishesh M., Hapangama D.K. (2019). Enriching Personalized Endometrial Cancer Research with the Harmonization of Biobanking Standards. Cancers.

[22] Romero I., Churruca C.M., Redondo A., Santaballa A., Calvo E., Ojeda B., Del Campo J.M., Laínez N., García-Martínez E., Romeo M. (2015). Early stage ovarian cancer clinical behavior according to FIGO 2014 Staging changes with a focus on IC subtype: Data from prospective GEICO registry. J. Clin. Oncol..

[23] Leskela S., Romero I., Cristobal E., Pérez-Mies B., Rosa-Rosa J.M., Gutierrez-Pecharroman A., Caniego-Casas T., Santón A., Ojeda B., López-Reig R. (2020). Mismatch Repair Deficiency in Ovarian Carcinoma: Frequency, Causes, and Consequences. Am. J. Surg. Pathol..

[24] Simeon-Dubach D., Watson P. (2014). Biobanking 3.0: Evidence based and customer focused biobanking. Clin. Biochem..

[25] Compton C. (2007). Getting to personalized cancer medicine: Taking out the garbage. Cancer.

[26] Hutter C., Zenklusen J.C. (2018). The Cancer Genome Atlas: Creating Lasting Value beyond Its Data. Cell.

[27] The Cancer Genome Atlas Research Network (2011). Integrated genomic analyses of ovarian carcinoma. Nature.

[28] Lehmann S., Guadagni F., Moore H., Ashton G., Barnes M., Benson E., Clements J., Koppandi I., Coppola D., Demiroglu S.Y. (2012). Standard preanalytical coding for biospecimens: Review and implementation of the Sample PREanalytical Code (SPREC). Biopreserv. Biobank..

[29] Moore H.M., Kelly A.B., Jewell S.D., McShane L.M., Clark D.P., Greenspan R., Hayes D.F., Hainaut P., Kim P., Mansfield E. (2011). Biospecimen reporting for improved study quality (BRISQ). J. Proteome Res..

[30] Te Yang H., Shah R.H., Tegay D., Onel K. (2019). Precision oncology: Lessons learned and challenges for the future. Cancer Manag. Res..

[31] Esteva-Socias M., Artiga M.-J., Bahamonde O., Belar O., Bermudo R., Castro E., Escámez T., Fraga M., Jauregui-Mosquera L., Novoa I. (2019). In search of an evidence-based strategy for quality assessment of human tissue samples: Report of the tissue Biospecimen Research Working Group of the Spanish Biobank Network. J. Transl. Med..

[32] Ferdyn K., Gleńska-Olender J., Witoń M., Zagórska K., Kozera Ł., Chróścicka A., Matera-Witkiewicz A. (2019). Quality Management System in the BBMRI.pl Consortium: Status Before the Formation of the Polish Biobanking Network. Biopreserv. Biobank..

[33] (2021). Horizon Europe, Work Programme 2021–2022. https://ec.europa.eu/info/funding-tenders/opportunities/docs/2021-2027/horizon/wp-call/2021-2022/wp-1-general-introduction_horizon-2021-2022_en.pdf.

[34] Kaye J., Heeney C., Hawkins N., de Vries J., Boddington P. (2009). Data sharing in genomics—Re-shaping scientific practice. Nat. Rev. Genet..

[35] Golubnitschaja O., Epma, Costigliola V. (2012). General report & recommendations in predictive, preventive and personalised medicine 2012: White paper of the European Association for Predictive, Preventive and Personalised Medicine. EPMA J..

